# Intracranial pressure monitoring in neurosurgery: the present situation and prospects

**DOI:** 10.1186/s41016-023-00327-2

**Published:** 2023-05-12

**Authors:** Chenqi He, Chubei Teng, Zujian Xiong, Xuelei Lin, Hongbo Li, Xuejun Li

**Affiliations:** 1 Department of Neurosurgery, Xiangya Hospital, Central South University, Changsha, Hunan 410008 People’s Republic of China; 2 Hunan International Scientific and Technological Cooperation Base of Brain Tumor Research, Xiangya Hospital, Central South University, Changsha, Hunan 410008 People’s Republic of China; 3 Department of Neurosurgery, the First Affiliated Hospital, University of South China, Hengyang, Hunan 421001 People’s Republic of China

**Keywords:** ICP, Cerebrospinal fluid, Zero drift, Wireless telemetering, Absorbable, Non-invasive

## Abstract

Intracranial pressure (ICP) is one of the most important indexes in neurosurgery. It is essential for doctors to determine the numeric value and changes of ICP, whether before or after an operation. Although external ventricular drainage (EVD) is the gold standard for monitoring ICP, more and more novel monitoring methods are being applied clinically.

Invasive wired ICP monitoring is still the most commonly used in practice. Meanwhile, with the rise and development of various novel technologies, non-invasive types and invasive wireless types are gradually being used clinically or in the testing phase, as a complimentary approach of ICP management. By choosing appropriate monitoring methods, clinical neurosurgeons are able to obtain ICP values safely and effectively under particular conditions.

This article introduces diverse monitoring methods and compares the advantages and disadvantages of different monitoring methods. Moreover, this review may enable clinical neurosurgeons to have a broader view of ICP monitoring.

## Background

ICP is the pressure produced by the cranial contents on the skull wall and is usually represented by Cerebrospinal fluid (CSF) pressure. The normal value of ICP ranges between 0.7 and 2.0 kPa (70–180 mm H_2_O) in adults and 0.5–1.0 kPa (50–100 mm H_2_O) in children. The pathological state of brain trauma, brain tumor, hydrocephalus and other diseases can lead to disorders in CSF circulation and venous reflux, resulting in corresponding ICP changes. The early clinical features of ICP change are crucial for the clinicians to further manage the patients. Unfortunately, in clinical practice, one of the biggest problems are how to judge the inchoate change of ICP. Clinical characteristics including headache, visual symptoms, tinnitus, and evidence of elevated CSF pressure on lumbar puncture do not perform well for continuous ICP monitoring. Thus, doctors are prone to supervise ICP by various monitors to improve clinical outcomes [1].

By continuous monitoring, clinicians can predict the clinical symptoms of the patient and take timely treatment measures to prevent adverse outcomes of neurocritical patients. The use of monitors enables clinicians to control ICP rather than wait for changes in ICP before taking corresponding measures. In addition, the monitoring and control of ICP can reduce secondary nerve injuries associated with morbidity and mortality [2].

Since Lundberg's landmark work in the late 1950s [3], various ICP monitoring technologies have been continuously developed and improved with the progress of science and technology. At present, the ICP monitors are classified into invasive and non-invasive types, and the invasive types are subdivided into wired and wireless. At present, the most widely used monitors are still the earliest wired ICP monitors, due to their high operation accuracy and use stability. However, complications such as intracranial infection, hemorrhage, and displacement limit the application of a wired ICP monitor [4–10]. With the emergence of new materials and the replacement of electronic components, wireless monitoring and non-invasive monitoring methods have gained popularity in clinical neurosurgery as they have fewer complications and contraindications than wired ICP monitors.

This paper compares the benefits and drawbacks of multifarious ICP monitoring, discusses the latest development of wireless ICP monitoring and its practical value in clinical practice, enumerates numerous kinds of non-invasive ICP monitoring techniques, and puts forward the future development trend of clinical ICP monitoring methods (Table 1).

**Table 1:** The comparison of different methods of ICP monitoring

ICP methods	Advantages	Disadvantages	Clinical practice
Wired ICP monitoring	1. Monitor continously 2. Ddirect contact with brain tissue, CSF, and other cranial contents 3. Able to drainage CSF 4. Simultaneously monitor other biochemical information 5. Perform CSF drainage at the same time	1. Complications,including infection, hemorrhage, sensor breakage, displacement,brain injury and so on 2. Zero drift and short using time 3. Need repeatedly operations	1. The gold standard of ICP measurement 2.most commonly used
Wireless ICP monitoring	1. Allow the patients to move freely 2. Continuous ICP measurements anywhere 3. Extend the duration of ICP monitoring and decrease the complication than wired ICP monitoring 4. Bioabsorbable ICP monitor no longer need removing the ICP sensor	1. Unable to drainage CSF 2. Novel ICP monitors need further study	Not used in clinic so far
Non-invasive ICP monitoring	1. No injury to the patient 2. Provide a valuable reference 3. Safe, quick, repeatable, easy to operate and relatively inexpensive 4. No complication 5. Be able to assess other cerebrophysiology condition	1. Difficult to obtain true and accurate ICP values with the current technology 2. Need professional trainning and judgment 3. Prone to subfective error 4. Patients with other diseases may also affect the evaluation of ICP 5. No consistent standard	As an auxiliary means to monitor ICP

### Invasive ICP monitoring

Invasive monitoring is a measurement method for the real-time monitoring of ICP by implanting a sensor into the brain. Compared with non-invasive monitoring technology, the significant merits of invasive monitoring technology are that its sensors are in direct contact with brain tissues, CSF, and other cranial contents to measure ICP. In addition, it can simultaneously monitor CSF pH, brain oxygen partial pressure, and other biochemical information. According to the implantation site of the monitoring probe, wired monitoring can be divided into intraventricular type, parenchymal type, epidural type, subdural type, and lumbar pressure monitoring.

ICP can also be measured through lumbar pressure monitoring although lumbar puncture is not recommended in most cases of high ICP. Monitoring ICP by lumbar puncture is not accurate and non-continuous. The value assessed by lumbar puncture may also not be equal to the intracranial ICP value due to hydrocephalus.

All the methods and principles of ICP monitoring have their merits and demerits. Considering the implant site, the main demerits of the brain parenchyma ICP monitors is that they only reflect the local pressure at the implant site, possibly making the readings misleading. Due to the pressure gradient throughout the brain, not every part operates under the same pressure. For intracerebroventricular ICP monitors, it is difficult to insert the catheter tip accurately when the patient has cerebral edema or ventricular shrinkage. After the catheter is inserted, brain tissue, blood clots, protein deposits, and other causes leading to catheter blockages can lead to inaccurate measurements. The presence of bubbles in the catheter may also cause unstable ICP data to be measured.

Furthermore, intraventricular catheters are not as safe as parenchymal catheters. Due to the characteristics of the epidural intracranial space, the epidural pressure is systematically higher than the intraventricular fluid pressure [11], resulting in inaccurate ICP measurements. Moreover, depending on the principles of different monitors, ICP monitors contain fiber optic, strain gauge, pneumatic (airbag), and fluid-based sensors [10, 12, 13].

### Fiber-optic sensor monitor (Camino ICP monitor) and pneumatic sensor ICP monitor (Spiegelberg ICP monitor)

The principle of the optical fiber ICP sensor can be summarized as follow: the output light is modulated by pressure or other physical parameters, thus causing mirror displacement. The Camino ICP monitor has been widely used in patients with intracranial diseases, since its initiation in neurosurgery in 1993 [14, 15]. After continuous testing and improvement, the optical fiber ICP monitor now features good sensitivity, accurate measurement value and competitive price. Moreover, fiber optic sensing systems are particularly suited for minimally invasive procedures, allowing precise point, multipoint, or distributed measurements without the need to increase the sensor size [16].

However, optical fiber monitors cannot solve the inherent drawbacks of wired monitors, such as vulnerability, long-term instability, inconsistency, and zero drift. Poor biocompatibility of metal components and high sensitivity to electromagnetic interference may also affect the use of fiber-optic sensors in clinical practice [16]. On the other hand, studies have also found that fiber optic technology is particularly suitable for developing “MR-compatible” sensors, because they are not affected by electromagnetic fields. The materials for manufacturing fibers do not interfere with the magnetic field inside the MR scanner, which is a key factor in maintaining the quality of the diagnostic information [17]. Since the fiber sensor is not a conductive material and has a power-on circuit assembly, it greatly reduces the adverse event caused by the electromagnetic interaction during the MRI scan or current leakage caused by defects in the device package [18].

The main complications of the Camino ICP monitors are infection, hemorrhage, fiber fracture, and displacement. Zero drift is the primary limitation of applying this optical fiber sensor because this type of micro-transducer cannot be recalibrated in situ. M. Galabert Gonz et al. had a mean drift of 7.3 ± 5.1 mmHg and found no significant correlation between zero drift and monitoring time [14]. In the study of Münch E et al. the actual average drift of the Camino monitor was 1.4 ± 5.3 mmHg, and the absolute average drift calculated from the absolute value of zero was 3.6 ± 4.1 mmHg [15]. The inaccuracy of the data is troublesome not only because clinicians cannot correct the zero drift but also because it is most likely to be found after extracting the monitor, which severely impacts the timely guidance for clinical treatment. On the other hand, the studies on the accuracy, operating characteristics and incidence of complications show that the use of Camino monitor provides a safe and reliable record for routine neurosurgical practice [15, 19].

As the representative of the pneumatic ICP monitors, the Spiegelberg ICP monitor is the first monitor that could be zeroed in situ. This monitoring system consists of an inflatable catheter with a balloon at the end, placed inside the brain parenchyma using a bolting system similar to other ICP monitoring devices. The ICP passes through the tube wall and is transmitted along the catheter to a pressure sensor within the electronic monitor, and the pressure sensor opens to the atmosphere once an hour to compensate for any zero drift [20].

The dominating advantage of the Spiegelberg pressure sensors is that they perform periodic automatic zeroing throughout the measurement period, and the latest Spiegelberg ICP monitor is conditionally compatible with the 1.5 T and 3 T MR equipment with good accuracy. Compared with other invasive monitoring methods, Spiegelberg monitors have characteristic good performance. Czosnyka et al. [21] compared the Camino with the Spiegelberg monitor and showed that the long-term zero drift of both probes were less than 0.7 mmHg, with the Spiegelberg monitor showing no temperature drift. Lang et al. [22] demonstrated that the absolute difference between the Spiegelberg reading and the EVD reading was less than ± 3 mmHg in 99.6% and less than ± 2 mmHg in 91.3% of cases. The Altman-Bland bias plot shows a good agreement between the Spiegelberg and EVD with a mean deviation of 0.5 mmHg. But as the ICP increases above 25 mmHg, the Spiegelberg exhibited a significant decrease of 10% in the number of reads. Yau et al. [23] showed a linear correlation between ICP measurement using a Spiegelberg ICP monitor and ICP measurement with an external ventricular drainage catheter. The results illustrate that the Spiegelberg ICP monitor has good practicality.

Some complications will inevitably occur during the use of wired ICP monitors. The incidence of adverse events such as infection, bleeding, and hardware failure associated with the use of wired ICP monitors ranged from 6 to 20%. In the study by Onhoff [24], 7% of the 152 patients with chronic hydrocephalus who underwent continuous ICP monitoring experienced minor complications, including 4 cases of accidental removal of ICP probes, 2 cases of failure to remove the probes that required surgical removal, and 2 cases of simple seizures, in the absence of trauma-related coagulopathy or brain swelling. Sorinola et al. [25] also evaluated the factors associated with the elevation of ICP-induced infection by EVD and identified 15 possible influencing factors, including age, gender, complicated infection, out-of-hospital catheter, catheter type, CSF leakage, CSF sampling frequency, diagnosis, catheterization time, ICP > 20 mmHg, flushing, multiple catheters, neurosurgery, decreased CSF and blood glucose during intubation. The adverse events related to the application of wired ICP have prompted the consideration of the development and application of wireless ICP monitoring.

### Wireless ICP monitoring

In 1967, Olsen and Atkinson simultaneously invented the wireless intracranial pressure monitor. Coincidentally, both of their proposed models for wireless ICP monitoring used telemetry to monitor ICP.

Olsen’s passive resonant sensor remotely monitors ICP by continuously measuring the size of the bubble [26], which will mechanically tune the resonant circuit and estimate the ICP change from the recorded waveform. The sensor is placed in a short, thin-walled glass tube with a volume of 0.06 mL with polyester film (ethylene terephthalate) ends of 6 mm diameter. The glass tube has a high Q distributed resonant circuit, and its frequency varies sensitively with the relative coil spacing. As pressure on the capsules forces these coils closer together, both the capacitance between the helices and their mutual inductance increase, reducing the resonant frequency of the configuration. The monitor then repeatedly scans the frequency of the inductively coupled oscillatory monitor fixed or mounted on the scalp and detects the electromagnetic energy absorbed by the transducer when it resonates through the intermediate tissue to record the intracranial pressure continuously. Finally, Olsen’s experiment proves that the modified system can record the change of 0.5 mm water column.

Atkinson’s “Miniature Passive pressure sensor” consists of a small section of glass tube wrapped in polyethylene, each end of which is connected to a polylactide film in a drum-shaped fashion [27]. Olsen’s and Atkinson’s monitors are not only similar in construction and form, but also in principle.

Since they have no external components, the wireless monitors have leinfection risk, which means this types of monitors can be used in patients for longer periods. The most prominent merit of wireless monitors is that patients are allowed freedom of movement, enabling continuous ICP measurements at home and potentially reducing the length and frequency of hospital stays. This not only improves patient compliance but also provides significant economic impact and time saving. Furthermore, extending the duration of ICP monitoring provides a theoretical possibility for long-term evaluation of CSF dynamics and thus provides an effective means for further study of ICP changes. Using a wireless monitor, the clinician can obtain ICP values without additional invasive procedures once the intracranial part is implanted, allowing the clinicians to observe the patient’s condition and assess whether the patient needs conservative treatment or further surgical treatment. For example, whether complicated hydrocephalus requires further shunt surgery, and whether patients with brain trauma at risk of cerebral herniation need further management.

A noteworthy advantage of wired monitors over wireless monitors is that certain types of intracerebroventricular ICP monitors can perform CSF drainage simultaneously. Nevertheless, analyses have shown that early CSF drainage does not improve the prognosis [28]. Lele’s analysis shows that the implantation of an ICP monitor within 72 h of admission without CSF drainage could reduce in-hospital mortality [29].

A dedicated ICP sensor is more accurate than measuring the ICP through an intracerebroventricular drainage system. Because the pressure signal obtained through a dedicated sensor is less prone to artifacts than that obtained through a fluid filling system. When a ventricular catheter is attached to a monitor, compression of the ventricle or blockage of the catheter can cause incorrect readings on the ICP monitor. Ideally, ICP monitoring systems and EVD devices should be independent so that the ICP monitoring system can still work properly when the drainage tube is removed, accidentally protruded or blocked.

### Piezoresistive wireless ICP monitor with telemetry (Codman, Raumedic Neurovent-p)

As mentioned earlier in this paper, in 1967, two teams invented the wireless ICP monitor using telemetry simultaneously. Since then, many other wireless systems have emerged, but the inevitable measurement uncertainties caused by technical limitations have impaired the development of wireless monitors. The basic principle of wireless ICP data transmission with telemetry is based on two inductively coupled resonant circuits, same as the original wireless ICP monitor. The external sensing device generates an electromagnetic field, and the inner coil capacitance circuit continuously oscillates. A flexible electrode in contact with the brain tissue makes up the capacitor. The distance between the capacitor electrodes varies with the intracranial pressure, leading to the frequency change of the resonant circuit. These influence the frequency of the sensing device and transform the electrical change to a signal, indicating the ICP [30].

**Fig. 1:**
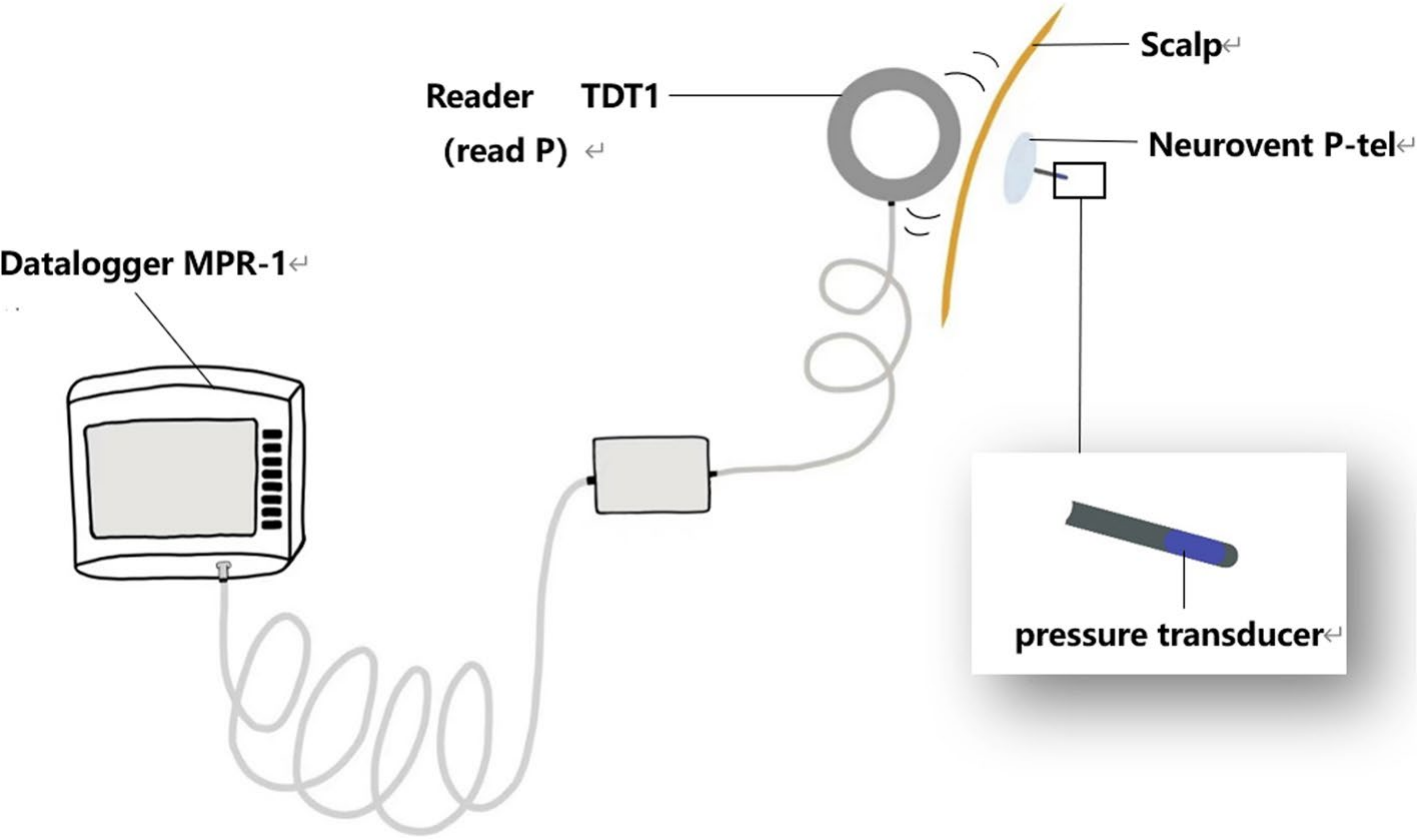
Schematic diagram of Neurovent-P-tel ICP monitor, drawing based on reference [30]. The pressure transducer (blue) is located at the end of a polyurethane catheter (30 mm in length, 1.67 mm in diameter). The circular ceramic housing (31.5 mm in diameter, 4.3 mm in height) contains a microchip (Neurovent P-tel). The antenna (card reader TDT1) is close to the shell, when the microchip is activated, then the TDT1 reading P is connected to a dedicated monitor (Datalogger MPR-1) to display and store transmitted ICP data

The following is a brief introduction of this type of ICP monitor using a telemetry device called Neurovent P-tel, launched by Raumedic in 2009 [30]. Passive implant, active antenna, and display storage unit are the three main parts of the ICP measurement system (Fig. 1). The pressure sensor is piezoresistive, consisting of several resistors, and doped into a flexible film in direct contact with the pulsating brain tissue. The stretching of the film, the length of the doping resistance, and the system’s resistance are persistently affected by the dynamic variation of ICP. These changes are recorded by a microchip (Neurovent P-tel), which converts these circuit values into ICP values. The power supply and ICP data transmission are based on radio frequency identification technology: the TDT1 reader activates the P-tel microchip by generating an oscillating electromagnetic field.

Freimann compared data obtained by the Neurovent-P-tel sensor with simulated riser measurements and found an average difference of 0.2 mmHg between the two technologies. These results indicate that the Neurovent P-tel sensor has high accuracy in measuring ICP over a large measurement range of 3–30 mmHg [31]. Pedersen et al. demonstrated that the wireless ICP monitor is safe for ICP monitoring in children and may reduce the number of invasive operation [32]. Caballero et al. demonstrated the reliability and practicality of the Neurovent-P monitor in making long-term clinical decisions [33]. Overall, the Neurovent P-tel strain gauge telemetry sensor has been shown to be effective in clinical applications (Fig. 1).

### Airbag wireless ICP monitor

Jiang et al. [34] designed an airbag wireless ICP monitor in 2019. It consists of an implantable ICP monitoring device under the scalp, an in vitro wireless data recorder and a patient data management computer. The ICP monitor consists of a pressure sensor, an ultra-thin airbag for pressure sensing, and a low-power dedicated system-on-a-chip (SoC) for data acquisition control and wireless transmission. The ICP device is implanted subcutaneously outside the skull. The absolute fluid pressure is measured by inserting a pressure-sensitive balloon into the intracranial space, including intracerebroventricular, parenchyma, epidural and subdural spaces. The absolute liquid pressure that is equal to CSF pressure is then transmitted wirelessly to a 416 MHz data logger, which is connected via a USB cable to a computer equipped with patient data management software. The ICP data can be viewed in real-time in the computer software.

This monitor has several features. First, the intracranial part of the device does not need to come into direct contact with CSF, reducing the risk of infection. Second, the monitor contains a dedicated system-on-a-chip consisting of a power management unit (PMU), an RF transmitter, workflow control logic, etc. Third, the device’s sensors include pressure sensors and temperature sensors. The pressure sensor measures the pressure of the gas in the shell cavity, and the pressure value will be very close to the intracranial pressure value of the place to be measured after selecting the appropriate material and thickness of the airbag. The temperature sensor is used to obtain the temperature outside the skull and under the scalp of the site to be measured. This temperature data is used to assist patient monitoring and treatment. Fourth, the telemetry circuits of ICP systems typically operate in the 2.4 GHz band, which can cause significant signal loss when radiofrequency signals pass through human tissues. Furthermore, 2.4 GHz telemetry is susceptible to measurement errors caused by interference from non-medical devices such as Wi-Fi routers or microwave ovens [35]. The frequency band used for wireless data transmission in this ICP monitor is 400–432 MHz, avoiding interference from other devices.

**Fig. 2:**
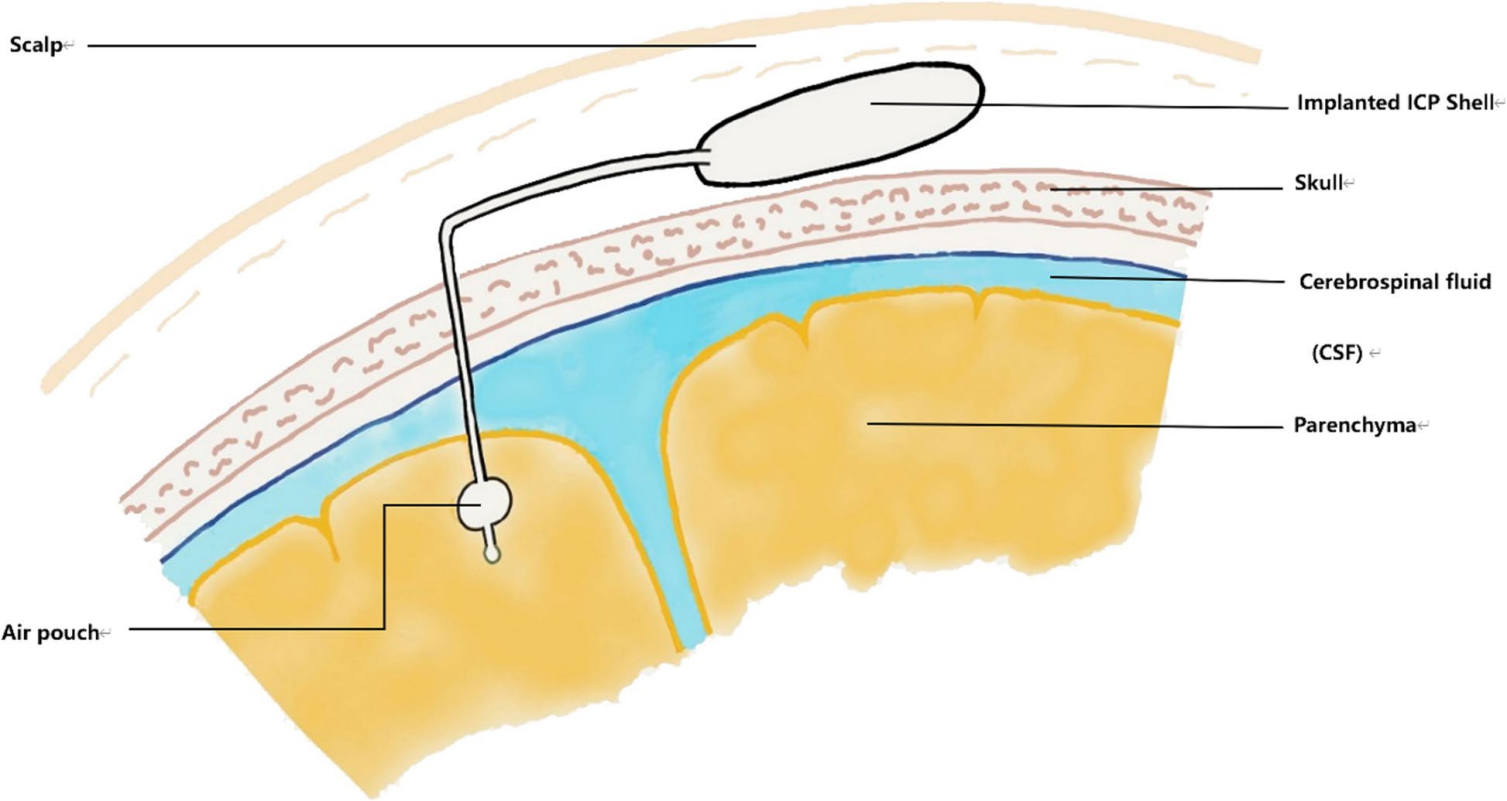
Schematic diagram of airbag ICP monitor, drawing with the contents for reference [34]

The ICP device has been tested in a liquid environment. The non-linear error is less than ± 0.4 mmHg in the full measurement ranges from − 20 to + 150 mmHg. However, this ICP monitor is unsuitable for brain parenchyma monitoring because changes in the balloon volume can cause serious brain tissue damage. By the time of writing this paper, there is still a lack of related animal experiments (Fig. 2).

### Bioabsorbable ICP monitor

The current wireless ICP monitoring devices in clinical use all require a second operation to remove the implant. When patients no longer need intracranial pressure monitoring, removing the ICP monitor requires a second operation, which can lead to secondary injuries and increase the patients’ psychological burden. Therefore, we are seeing the rise of studies on bioabsorbable intracranial pressure monitors that do not require secondary surgery.

In 2014, Luo et al. [36] invented a functional pressure sensor based on a passive resonance mechanism made entirely of biodegradable materials. The wireless intracranial pressure sensor uses a zinc (Zn)/iron (Fe) bilayer as the conductor and biodegradable polymers poly (L-lactide) (PLLA) and poly (caprolactone) (PCL) as the dielectric and structural materials. These materials were chosen because Zn has reasonable electrical properties for high Q elements and is also a biocompatible metal. PLLA is used as a packaging and pressure-sensitive plate material due to its good mechanical properties, while PCL is used as bonding and sealing material due to its low melting temperature (about 61 °C).

The pressure sensor consists of a sensing chamber surrounded by two electrodes forming a variable capacitance and connected to an inductor coil. The inductor coil acts as the basic component of the resonant sensor and provides a device for magnetically coupling the sensor to the external coil. When pressure is applied to the sensor, the gap between the two capacitive electrodes decreases and the capacitance value increases. The resulting pressure-induced change in resonant frequency can be measured wirelessly using an external coil.

Current experiments have shown a sensitivity of 39 kHz/kPa measured in the 0–20 kPa pressure range in air and brine. After being immersed in normal saline for 20 h, the sensor remained stable and worked normally for 86 h. This ICP monitor is a very promising sensor, with biodegradable polymer as the packaging material and biodegradable metal as the conductor.

In 2017, Kang et al. [37] published an article introducing a new absorbable wireless ICP monitor. The main component of this instrument is the intracranial receptor part and the external data transmission of the miniature wireless potentiate. The main structures of the intracranial sensor are poly (lactic acid and glycolic acid) copolymer membrane, snake-shaped silicon nanofilm, and silica overlay. The sensor quantitatively captures the mechanical behavior of the system through three-dimensional finite element analysis. At the same time, the silicon nanofilm acts as the flow sensor of both the heating element and the temperature sensor and a pH sensor that relies on electricity. The device is unique in that its intracranial sensors are fully bio-absorbable, and they dissolve completely into biocompatible substances when immersed in an aqueous solution, including biological fluids such as CSF.

In addition, the article mentions another feature of this device, which is the measurement of other parameters of interest with a variety of simple modifications. For example, a motion sensor using poly lactic-co-glycolic acid (PLGA) cantilever to test mass; A temperature sensor using a temperature-dependent resistance of a silicon nanofilm element separated from the cavity structure. The silicon nanofilm acts as both a heating element and a temperature sensor, such as a flow sensor. In vivo and in vitro experiments have demonstrated accurate measurements of pressure, temperature, motion, flow, thermal properties and pH, and may extend to biomolecular binding events.

In 2019, Shin et al. [38] developed an ultra-thin silica film that can be used as a bioabsorbable encapsulation layer to enable ICP and temperature sensors in rats to operate stably over 25 days. This ultra-thin silica film can be used as a bioabsorbable encapsulation layer, which dissolves in a simulated biological fluid at physiological temperatures at a rate of several hundredths of a nanometer per day, producing silicic acid as the final solution product. This product has been tested in vitro and in vivo in mice. Its advantage over the absorbable wireless ICP monitor invented by Kang mentioned above is that it can be used for a long time.

### Introduction of non-invasive ICP monitoring and its comparison with invasive ICP monitoring

In the clinic, some inspection items that are typically used to examine other diseases can also be utilized to monitor ICP, such as ultrasonography, computed tomography (CT), magnetic resonance imaging (MRI) and so on. Additionally, non-invasive ICP monitoring methods can be classified into four main categories based on their different principles: fluid dynamics, otology, ophthalmology, and electrophysiology [10].

In the following section, we will focus on representative non-invasive ICP monitoring methods that are widely used in clinical practice and compare their advantages and disadvantages with invasive ICP monitoring.

### ICP monitoring by ultrasonography

Ultrasonography can be used to evaluate the anatomy and pathology of the brain and to evaluate cerebral circulation by analyzing the blood flow velocity. This means that various ultrasound techniques can be used to track changes in ICP, such as optic nerve sheath diameter (ONSD),ventricular width measurement, midline offset, arterial resistance, and so on [39].

Furthermore, ultrasonography is a safe, repeatable, and non-invasive technique that can be used in various environments, including out-of-hospital, emergency room, surgical operating room, intensive care, and general wards [40, 41]. A specially trained physician can indirectly monitor ICP by performing a relatively simple and quick bedside ultrasound. Additionally, ultrasonography may compensate for a bedside neurological physical examination, an examination requiring patient transport, or an invasive examination in some clinical settings.

Ultrasonography, especially transcranial doppler (TCD), can help clinicians determine whether patients need further investigation, such as CT, computed tomography angiography (CTA), MRI, invasive ICP monitoring, or other surgical interventions [42]. This technique is useful for patients in unstable conditions, with contraindications to lumbar puncture, and patients who refuse surgical implantation of ICP monitors.

The advantage of brain ultrasound for ICP monitoring is evident in patients with diseases that do not typically require surgically implanted ICP monitors, such as cerebral malaria, persistent epilepsy, mild or moderate brain trauma, and brain tumors. Therefore, ultrasonography and invasive ICP monitoring techniques complement each other in clinical practice.

### ICP monitoring by optic nerve sheath diameter (ONSD)

**Fig. 3:**
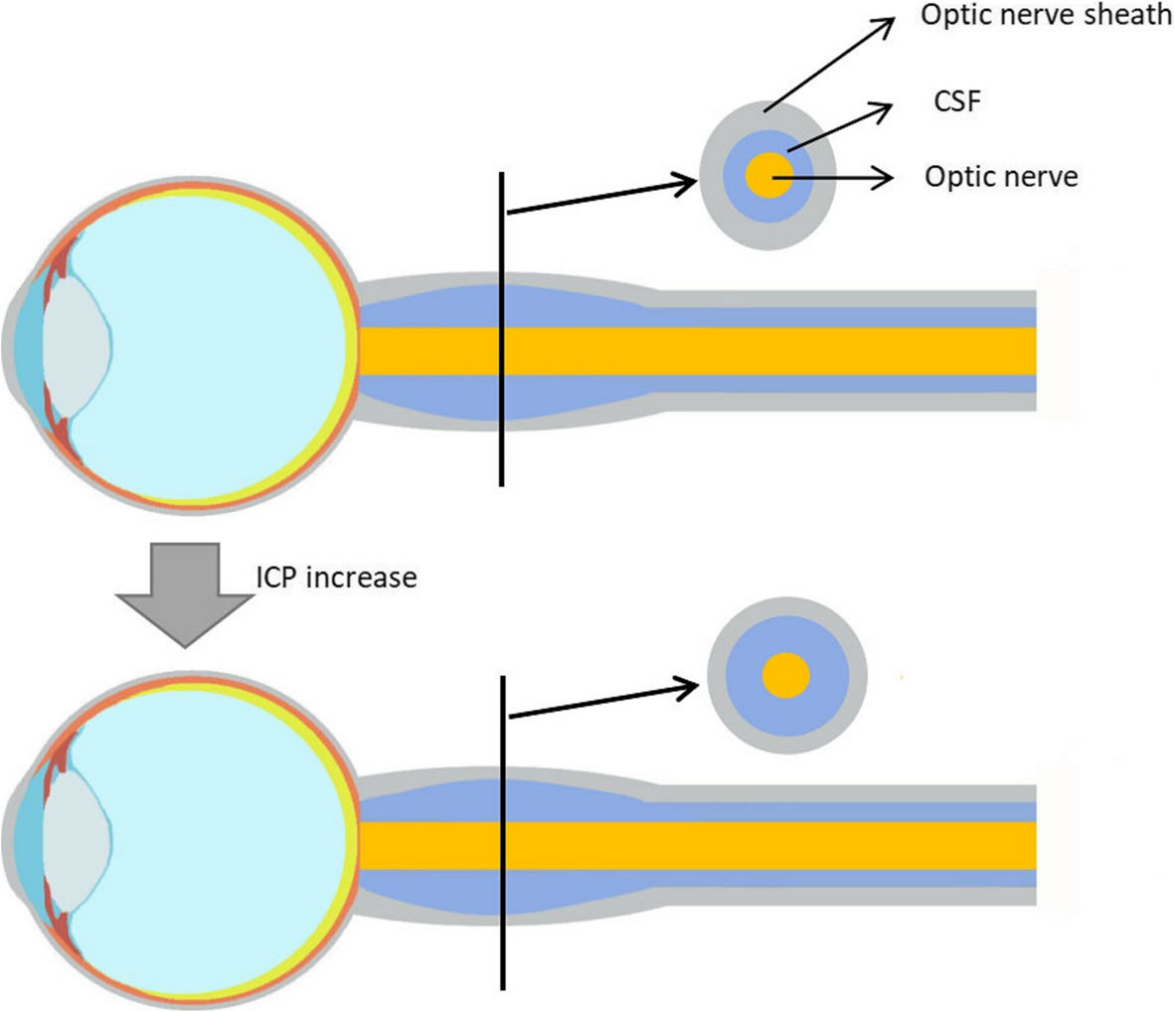
A simple schematic of ONSD. The change in ICP due to changing CSF results in the corresponding changes to the posterior globe pressure

The space between the optic nerve and the optic sheath is adjacent to the underlying cavity of the subarachnoid such that the change in ICP due to changing CSF results in the corresponding changes to the posterior globe pressure. Hence, the change in ONSD reflects the change in ICP (Fig. 3) [2, 43].

Some studies demonstrate that ONSD displayed satisfied consistency with invasive ICP monitoring. Dubourg’s study with an ONSD monitoring method has shown that the consolidated sensitivity of ICP is 0.90, having a combined specificity of 0.85 (95%CI 0.73–0.93), and summary receiver-operating characteristic (SROC) curve 0.94 (95%CI 0.91–0.96) [43]. Padayachy et al. have found that the optimal diagnostic accuracy of ICP ≥ 20 mmHg is 5.5 mm with a sensitivity of 93.2% and specificity of 74% [44].

The above research results show that ONSD measurement provides a decent estimate of the ICP, but its interpretation is limited by the lack of ONSD unified critical value standards. Moreover, results should be interpreted with caution for patients with ophthalmic diseases, including optic neuritis, optic nerve injury, optic ginseng cyst, eyelid tumor, eye inflammation, nodular disease, GRAVES disease, and others [45].

In addition, the relationship between ONSD and ICP is not uniform between individuals; a substantial ICP reduction can lead to minor ONSD changes in some and vice versa in others [46]. Moreover, there is a large margin of error in this type of ICP measurement due to the relatively small increase in ONSD, inducing low specificity and many false positives.

The ONSD technique uses ultrasound and is a helpful bedside method for monitoring ICP. It can determine whether the patient needs to perform further aneurysm imaging and screen patients requiring invasive ICP monitoring. All in all, ONSD is considered a non-invasive and reliable means of monitoring ICP in adults [47–49]. Studies have shown that among all the ultrasound ICP monitoring methods, ONSD remains the most reliable parameter [50].

### ICP monitoring by transcranial doppler (TCD)

TCD is a technology that evaluates the cerebrovascular kinetics through cerebral blood flow velocity, and is a non-invasive and portable technology [51]. Qualitative and quantitative TCD monitoring of the ICP is mainly carried out by monitoring the frequency spectrum and waveform changes in the middle cerebral artery and basilar artery. The applicability of TCD in ICP evaluation was determined by observing the changes of TCD parameters, such as cerebral blood flow waveform or pulse index. Using TCD to monitor ICP is mainly based on an approximate semi-quantitative relationship between cerebrovascular dynamics and ICP. Thus, TCD can monitor ICP only when the changes occur in arterial vessels.

However, the accuracy of TCD monitoring of ICP may be lower if the ICP changes are caused by CSF circulation disturbances or increased brain parenchymal volume [52]. Furthermore, TCD detection results are largely dependent on the experience and skill of the operator. The operator must manually position the probe to obtain measurements along the vascular axis by detecting the maximum velocity position. Individual anatomical differences also create a challenge in determining an accurate ICP value.

Pradeep R et al. analyzed the TCD results before and after the patients underwent lumbar puncture with CSF opening pressure monitoring, and drew the conclusion that TCD-derived peak can be used for management, prognostication, and follow-up in patients with idiopathic intracranial hypertension [53].

It is worth mentioning that TCD can evaluate the brain’s automatic adjustment function, providing essential data for treating severe brain injury patients [54]. Besides, the American Heart Society SAH Treatment Guide recommends using TCD to monitor the development of arterial vasospasm as a class IIA level B evidence [55]. In the clinical setting, TCD monitoring is not limited to the simple estimation of ICP. Also, it can be used to assess the cerebrovascular condition, cerebral blood flow, brain autoregulation function, and other three-dimensional aspects to optimize decisions for patients at the same time.

### ICP monitoring by MRI

The principle of monitoring ICP using MRI involves deriving pressure changes during the cardiac cycle from the CSF pressure gradient waveform calculated from the CSF velocity. The variation of intracranial volume is determined by the instantaneous difference between arterial blood inflow, venous blood outflow, and CSF inflow and outflow from the top of the skull. Elasticity is derived from the ratio of the measured pressure to volume change. The mean ICP value is obtained from the linear relationship between intracranial elasticity and ICP [56, 57].

Galperin et al. [58] calculated the ICP value by the elastic index and proved the sensitivity of this method is sufficient to distinguish normal and elevated ICP. Rambha Burman et al. showed that MRI monitoring of ICP is positively correlated with invasive ICP monitoring. The invasive method ICP value was, on average, 2.2 mmHg higher than the MRI monitoring ICP [56].

In addition to establishing the relationship between intracranial elasticity and ICP through MRI technology, MRI can also determine the high and low of ICP from other aspects. For example, Long et al. [59] obtained CSF dynamic parameter values using phase-contrast MRI technology, built a non-invasive ICP function prediction model, and verified the correlation between the change of midbrain aqueduct diameter and the change of ICP.

The biggest problem with constructing the ICP function by imaging is that none can provide an exact ICP value. The results inevitably include false negatives and false positives. The evaluation of modeling parameters is affected by multiple factors, such as whether the patient's disease affects the CSF dynamics, the congenital anatomical variation between patients, the heterogeneity of the subjects, and so on. Therefore, before popularizing MRI monitoring of ICP, we need more large-scale research to further verify this method.

In addition, there are other problems to this technique, such as the expensive cost of MRI and the time-consuming and labor-intensivenature of the process, which may make it impractical for cases requiring immediate and continuous ICP monitoring.

### Other non-invasive ICP monitoring

In addition to the non-invasive ICP methods introduced above, other new monitoring methods are constantly emerging in clinical practice. For example, two depth transorbital doppler (TDTD) uses extracranial pressure to estimate ICP. The principle is that when the external pressure applied to the ophthalmic artery is equal to ICP, the blood flow waveforms of the extracranial and intracranial segments of the ophthalmic artery are equal [60].

M Bodo et al. [61] studied the relationship between rheoencephalography (REG) and ICP and concluded that the area under the subject operating characteristic curve in animal models was 0.9481 and 0.9335, respectively, indicating that REG can reflect ICP well. Furthermore, electroencephalogram changes [62, 63], visual evoked potential [64], neurological pupil index (NPI) [65], electrical impedance tomography (EIT) [66], near-infrared spectroscopy [67], and cochlear microphonic potential [68] are all associated with ICP.

Emerging technologies such as, venous opthalmodynamometry, tympanic membrane displacement [69, 70], tissue resonance analysis, tonometry, acoustoelasticity, distortion product oto-acoustic emissions, anterior fontanelle pressure monitoring, skull elasticity, and jugular bulb monitoring are also appearing. Nicolas Canac et al. have evaluated and graded the performance of various non-invasive ICP monitoring methods (Table 2) [10].

**Table 2:** Non-invasive ICP monitoring methods

Principles	Methods	Advantages	Disadvantages
Fluid dynamics	TCD 51–55	1. Get the cerebral vessel condition, cerebral blood flow and other parameters 2. NIRs use for the earlier recognition traumatic brain injury secondary insults	1. Pathological vessels and CSF will affect the result 2. Different processing Models will get different results 3. There is plenty variation in NIRS techniques and the reference measurements
	TDTD 60		
	Dynamic MRI 56–59		
	Near-infrared spectroscopy 67		
	Jugular bulb monitoring		
Otology	Tympanic membrane displacementw 69,70	CM measurements have been routinely used for decades in audiology	1. TMD complications, long-lasting intubation may resulting auditory-tube dysfunction and insufficient middle-ear ventilation or effusion 2. TMD variability varies considerably due to spontaneous pulsing and cochlear aqueduct 3. TMD limited by daily safe noise exposure limits and sedative drugs 4. Need exclude the abnormal transmission of sound through the ear
	Cochlear microphonic potential (CM) 68		
	Distortionproduct oto-acoustic emissions		
Ophthalmology	ONSD 43–50	1. Only need commom ophthalmic examinations 2. NPI may predict patient prognosis	1. Patients with ophthalmic diseases will influce the measurement result 2. Individual differences
	Neurological pupil index (NPI) 65		
	Spontaneous venous eye measurement		
	Venous opthalmodynamometry		
	Tonometry		
Electrophysiology	Rheoencephalography (REG) 61	1.Visual evoked potential can measure the pressure of the left and right compartments separately 2.The measurement results of EIT do not depend on the operator's experience	1.Exist time delay between EEG and ICP 2.Visual evoked potential influced by anatomic injury of the visual pathway 3.Disturbed by some drugs, severe acidosis, liver func-tion damage, sedation, and anesthesia
	Electroencephalogram (EEG) 62,63		
	Electrical impedance tomography (EIT) 66		
	Visual evoked potential 64		
Other	Tissue resonance analysis	Innovative technology	Lack of clinical experiment need further research
	Acoustoelasticity		
	Anterior fontanelle pressure monitoring		
	Skull elasticity		

At present, an increasing number of non-invasive methods of ICP monitoring are emerging in clinical practice, but intraventricular or parenchymal ICP monitoring is still considered the gold standard of ICP measurement. Non-invasive technology cannot replace invasive monitoring yet [2, 10, 45, 52, 71]. With the progress of science and technology, there is no doubt that non-invasive ICP monitoring technology will continue to develop, but it is difficult to obtain true and accurate ICP values with the current technology. Improvement of accuracy and precision is an urgent problem to be solved in non-invasive ICP monitoring.

Most non-invasive technologies require specific training for medical staff. The differences in the performance of various organizations, operating processes, and operators will directly affect the evaluation of ICP. In addition, patients with other diseases may also affect the evaluation of ICP. Although non-invasive ICP monitoring cannot replace invasive ICP monitoring at present, non-invasive methods can still provide a valuable reference for doctors to guide clinical treatment. It remains an effective and safe alternative to invasive ICP monitoring, especially in patients where invasive ICP monitoring is contraindicated, too risky, or difficult to perform [10, 72].

Moreover, analysis has shown that patients who maintain a specific ICP using invasive ICP monitors are not shown to have better outcomes than those based on imaging and clinical tests [73]. Therefore, even if non-invasive ICP monitoring cannot obtain an accurate ICP value, it can still be used as an auxiliary evaluation method to assist clinical treatment.

## Conclusion

In summary, an ideal ICP monitor for clinical use requires the following characteristics: ease of use, accuracy, reliable readings, and a low risk of infection, prolapse, bleeding, and rupture. In the future, the development of ICP monitoring methods will aim to be wireless, absorbable and minimally invasive. Furthermore, with the improvement of various non-invasive technologies, it is possible that non-invasive ICP monitoring will replace invasive monitoring and become the mainstream development trend in the future.

## Data Availability

Not applicable.

## Abbreviations

ICP: Intracranial pressure
CSF: Cerebrospinal fluid
ONSD: Optic nerve sheath diameter
TCD: Transcranial doppler
CT: Computed tomography
CTA: Computed tomography angiography
MRI: Magnetic resonance imaging
REG: Rheoencephalography
TDTD: Dual-depth transorbital Doppler
NPI: Neurogenic pupil index
EVD: External ventricular drainage
